# Role of *Actinomyces* spp. and related organisms in the development of medication-related osteonecrosis of the jaw (MRONJ): Clinical evidence based on a case series

**DOI:** 10.1556/1886.2023.00041

**Published:** 2023-12-01

**Authors:** Zsanett Kövér, Ágnes Bán, Márió Gajdács, Beáta Polgár, Edit Urbán

**Affiliations:** 1 Department of Dentistry, Oral and Maxillofacial Surgery, Medical School, University of Pécs, Tüzér u. 1., 7623 Pécs, Hungary; 2 Department of Oral Biology and Experimental Dental Research, Faculty of Dentistry, University of Szeged, Tisza Lajos krt. 64-66., 6725, Szeged, Hungary; 3 Department of Medical Microbiology and Immunology, Clinical Center, University of Pécs, Szigeti út 12., 7624, Pécs, Hungary

**Keywords:** Actinomyces spp., Schaalia spp., medication-related osteonecrosis, MRONJ, oral surgery, anaerobic bacteria

## Abstract

Medication-related osteonecrosis of the jaw (MRONJ) is an increasingly common consequence of antiresorptive treatment, which often leads to the development of necrotic exposed bone surfaces with inflammatory processes affecting the jawbone. Although the development of MRONJ is often associated with the inflammatory response or infections caused by the colonizing members of the oral microbiota, the exact pathogenesis of MRONJ is still not fully understood. In the present paper, we aimed to provide additional, microbiological culture-supported evidence, supporting the “infection hypothesis” that *Actinomyces* spp. and related organisms may play an important pathogenic role in the development of MRONJ and the resulting bone necrosis. In our case series, all patients presented with similar underlying conditions and anamnestic data, and have received antiresorptive medications (bisphosphonates or a RANK ligand (RANKL) inhibitor) to prevent the occurrence or progression of bone metastases, secondary to prostate cancer. Nevertheless, a few years into antiresorptive drug therapy, varying stages of MRONJ was identified in the mentioned patients. In all three cases, quantitative microbiological culture of the necrotic bone samples yielded a complex microbiota, dominated by *Actinomyces* and *Schaalia* spp. with high colony counts. Additionally, our followed-up case series document the treatment of these patients with a combination of surgical intervention and long-term antibiotic therapy, where favourable clinical responses were seen is all cases. If the “infection hypothesis” is valid, it may have significant consequences in the preventative and therapeutic strategies associated with this disease.

## Introduction

Medication-related osteonecrosis of the jaw (MRONJ) has been defined as a denuded bone surface area in the maxillo-facial region, which does not heal for two months after identification by a healthcare-professional, in a patient, who previously received specific medications, but not radiotherapy [1]. As a clinical entity and diagnosis, MRONJ was first described in 2003 as “induced avascular necrosis” of the jaws, which was associated with pamidronate or zoledronate therapy, leading to jaw pain, exposed bones, abscesses and osteomyelitis in the mandible or in the maxilla [2]; for this reason, the initial term to describe the condition was bisphosphonate related osteonecrosis of the jaw (BRONJ) for almost a decade [3]. Chemically, bisphosphonates (initially called dysphosphonates) are stable analogues of inorganic pyrophosphates (PPi), that affect bone resorption and remodeling mechanisms, by inhibiting osteoclast formation, activation and their attachment to the bone, in addition to promoting their apoptosis [4, 5]. The first derivative to be introduced in the clinical practice was etidronate in the 1960s [6]. Currently, bisphosphonates have a wide range of indications, including post-menopausal osteoporosis, Paget's disease, myeloma multiplex, and breast and prostate metastases in the bones, where they considerably increase the quality of life in affected patients [7]. The rationale for their use is due to their ability to prevent the occurrence of pathological fractures, to control hypercalcemia, to decrease bone pain, and to prevent metastasis formation and intraosseal tumor enlargement in malignant tumors with bone metastases [8, 9]. Due to their efficacy in the above mentioned conditions, bisphosphonates have become popular therapeutic modalities, however, this has corresponded to the more common detection of adverse events associated with their use [10]. As the binding affinity of bisphosphonates to the bone is considerable, they accumulate at the sites of otherwise high osteoclast activity [11]. This underpins why osteonecrosis occurs most often in the jaw, as these bone have a higher remodeling rate than others, therefore are more susceptible to the effects of bisphosphonates [12]. As a result, with no bone formation and bone resorption, the old bone survives, the associated capillary network is maintained, which leads to avascular necrosis of the jaw [13]. The slowed down and altered wound healing processes, mucosal insults and their delayed epithelial closures will lead to chronic infection and necrosis of the bone, which may be further aggravated by bacterial infections [14]. However, the exact mechanisms corresponding to why jawbones are most commonly affected with bone necrosis secondary to bisphosphonate therapy remain unclear.

In 2014, a proposal by the American Association of Oral and Maxillofacial Surgeons (AAOMS) resulted in a classification and guideline change for this condition from BRONJ to MRONJ, as cases of bone osteonecrosis were detected in patients receiving medications other than bisphosphonates [15]. These medications included other antiresorptive drugs with better pharmacokinetic properties (such as the receptor activator of nuclear factor kappa beta-ligand [RANK-L] inhibitor denosumab), and inhibitors of angiogenesis, like bevacizumab or sorafenib [16]. The current clinical classification system for MRONJ defines four different disease presentations, based on clinical severity: in Stage 0, patients present with atypical symptoms without a denuded bone surface (e.g., mild pain in the in the jaws and pain referred to temporomandibular joints, mild pain in the maxillary sinuses), spontaneous tooth loss, and extra- and intraoral swelling. Stage I is characterized by an asymptomatic patient, whereas, on clinical examination, an exposed and necrotic bone surface may be observed with fistula, but without pain and inflammation. In Stage II, exposed and necrotic bone surfaces may be observed with fistula, with or without purulent drainage, erythema and pain. Finally, at Stage III, in addition to the characteristics of Stage II, extraoral fistula, the occurrence of pathological fractures, and necrosis extending beyond the the alveolar bone may also be observed [15–17]. Based on the therapies received and the underlying conditions of the individual, patients may be classified as low-risk and high-risk for developing MRONJ. For example, the duration and the dose of antiresorptive treatment, and the method of administration (i.e. intravenous administration of bisphosphonates increases the risk by 100–1000-fold) are all relevant when determining MRONJ risk [10, 18]. Advanced age, the presence of other underlying conditions (e.g., Type II diabetes) affecting tissue healing and circulation, comorbidities affecting the patient's immune status, simultaneous use of other treatments (e.g., steroids, immune suppressants, estrogen receptor modulators, radiation therapy), poor oral hygiene and lifestyle factors (e.g., alcohol, tobacco consumption) should also be considered risk factors [18, 19]. Finally, it has been described that some polymorphisms in farnesyl pyrophosphate synthase and CYP2C8 genes, result in a genetic predisposition for BRONJ in multiple myeloma patients [20]. Depending on MRONJ stage and patient characteristics, treatment modalities may range from conservative antibiotic therapy to surgical resection of the jawbone [15–17]. Complications and sequelae of the disease cause a considerable decrease in the quality of life for the affected patients, however, due to the fact that patients often do not attend regular dental check-ups, the lesions are commonly only being identified in their advanced stages [21].

The human oral cavity is colonized by a diverse community of bacterial species, which are major contributors to homeostasis and disease for oral and systemic health [22]. According to some estimates, there may be over 700 bacterial species co-existing in various anatomical surfaces and in the oral biofilm, the majority of which are strict anaerobes [23]. Members of the *Actinomyces* genus are Gram-positive anaerobic rods, which are one of the most relevant microorganisms among the “early colonizers” of the oral cavity [24, 25]; they are ubiquitously found in the dental biofilms, and have important roles in the development of dental caries and other manifest dental infections (e.g., root canal and periapical infections) [26]. Members of the *Actinomyces* spp. have recently undergone extensive taxonomic revisions (due to the introduction of 16S rRNA sequencing results), leading to the reclassification of many clinically-relevant species (e.g., *A. odontolyticus*, *A. meyeri*, *A. pyogenes*) towards the more heterogeneous group of *Actinomyces*-like organisms (ALOs; e.g., *Schaalia odontolytica*, *Schaalia meyeri*, *Truperella pyogenes*) [26–28]; nonetheless, all these species may still be important causative agents of cervicofacial actinomycosis, a chronic granulomatous condition, which often leads to severe tissue destruction [25, 29]. The diagnosis of actinomycosis is often delayed due to the initial non-specific symptoms of the disease, while its treatment frequently requires surgical intervention, drainage and long-term antibiotic therapy [26, 28].

As a result of long-term antiresorptive treatment, bone metabolism in the jawbones is reduced, which – together with mucosal damage and impaired tissue repair mechanisms – leads to exposed bone surfaces in the oral cavity [30]. Although the development of MRONJ is often associated with the inflammatory response and/or infections caused by the colonizing members of the oral microbiota, the exact pathogenesis of MRONJ is still not fully understood [29, 31]. Co-aggregation, cooperations involving nutrient supply and immune evasion among microorganisms in the oral biofilm may be crucial in the pathogenesis of osteonecrosis, as these anaerobic microorganisms may bind to the collagen fibers in bone forming polymorphonuclear aggregates and bacterial biofilm, and facilitate bone resorption and tissue destruction [32]. However, it is still unclear whether the development of osteonecrosis is primarily caused by antiresorptive drug therapy (and oral bacteria are present only as colonizers in the necrotic bone) or whether bisphosphonates facilitate access to oral microorganisms – especially *Actinomyces* spp. and ALOs – to cause infection that results in osteonecrosis; the latter concept is termed the “infection hypothesis” for MRONJ [33, 34]. Nevertheless, the number of studies involving microbiological analyses corresponding to the exposed bone sites and necrotic bone tissue are limited. In these reports, the most common microorganisms in the exposed bone sites were *Actinomyces* and related species, forming co-aggregated with *Eikenella*, *Fusobacterium,* Gram-positive anaerobic cocci (GPAC), and *Veillonella* species, in patients with MRONJ and infected osteoradionecrosis (IORN) [35–37]. In addition, studies often report the presence of sulfur granules in deeper tissue and drainage areas, which supports the diagnosis of actinomycosis in MRONJ [38].

In the present paper, we would like to provide additional clinical evidence to this field, supporting the hypothesis that MRONJ may be considered a bisphosphonate-induced jaw infection. Herein, we present a documented and followed-up case series involving three patients, with similar medical histories and treatment characteristics, where the dominant presence of *Actinomyces* spp. and ALOs was confirmed in MRONJ-associated necrotic bone lesions by the means of quantitative microbiological culture methods. Additionally, after the established diagnoses, we describe the therapeutic protocol utilized (including surgical treatment and antibiotic therapy), which has resulted in the clinical cure of the patients involved.

## Case series

### Case 1

**Fig. 1.**
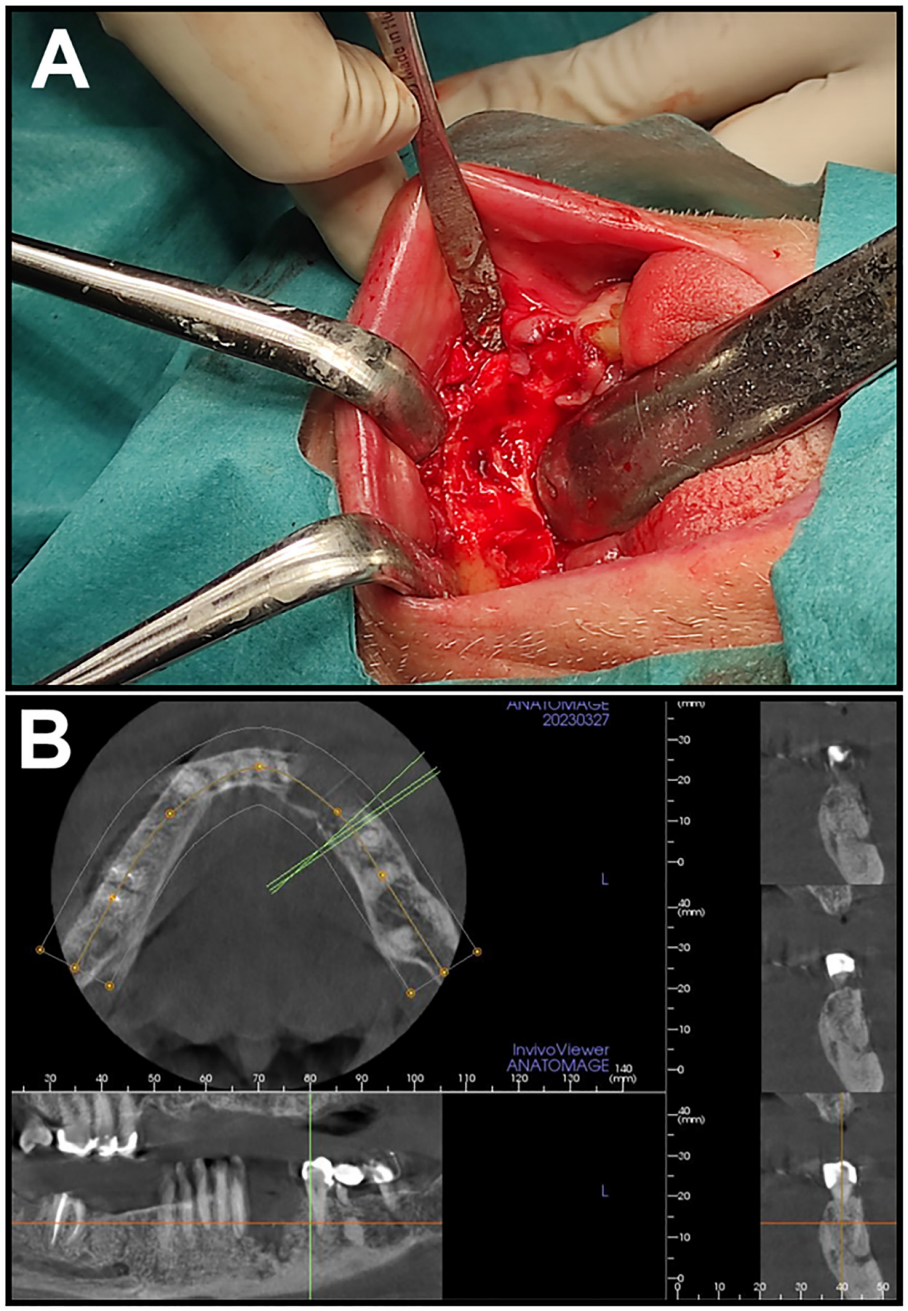
Clinical images corresponding to Case 1 **A:** Clinical presentation of the patient during surgery; **B:** Cone-beam CT (CBCT) image of patient before the surgical intervention

A 87-year-old male patient underwent transurethral prostatic resection (TURP), due to complaints of dysuria. TURP sampling confirmed a diagnosis of prostate adenocarcinoma presenting with bladder neck infiltration (with a Gleason score 7 (3 + 4)), for which, surgical therapy was chosen as primary treatment, followed up by endocrine deprivation therapy (ADT) and radiation treatment (81 Gy) for 6 months. Within two years of the initial diagnosis, scintigraphy has shown bone metastases in the right ilium, and in the thoracic and lumbal region of the spine, therefore intravenous (i.v.) zoledronic acid therapy was initiated. After two years, a control scintigraphy has identified isotopic enrichment in the left mandible (in the frontal and premolar region), however, it was attributed to dental causes. Control scintigraphies showed regression and stagnation of the patient's bone metastases, no progression was identified. On the other hand, isotopic enrichment in the left mandible was detected once again, and has become permanent in subsequent scintigraphies. No intra-oral pathological signs or symptoms associated with MRONJ or other complaints were noted during examination. One year after the inital detection of the isotopic enrichment in the left mandible, i.v. zoledronic acid therapy was switched to once-a-month i.v. denosumab treatment. 40-months afterwards, the tooth 3.4 was extracted at another institution; five months after the tooth extraction, the patient presented at the Department of Dentistry, Oral and Maxillofacial Surgery, and was treated with a 7-day course of i.v. amoxicillin 2 × 1 mg, as Stage 1 MRONJ was detected in the post-extraction area. After the one-week antibiotic therapy, the patient was symptom-free. No denuded bone surfaces were detected at the time of diagnosis, nor were they detected at the monthly follow-ups. After four years, denosumab therapy was stopped per request of the patient, as he could not tolerate the adverse effects of the medicine; based on control scintigraphies, no progression in the patient's bone metasases occurred. One year after the cessation of denosumab therapy, the patient was diagnosed with Stage 2 MRONJ during a dental check-up, in the post-extraction area and the left front and premolar region of the mandible. On multiple occasions, pus could be detected around the necrotic area. Surgical treatment was decided upon instead of conservative therapy. During the surgical procedure, the necrotic surface was removed, and the affected area was covered with platelet rich fibrin (PRF) and suture (Fig. 1). Under sterile conditions, a small fragment of the necrotic bone was taken from the surgical site, placed in 1 mL sterile, pre-reduced brain-heart infusion (BHI) broth (with a pH adjusted to 7.2; Oxoid, Basingstoke, United Kingdom), and sent for processing at the Department of Medical Microbiology and Immunology, where culture was initiated immediately. Traditional quantitative culture – under strict anaerobic conditions – was carried out, and after 5–7 days of incubation, *S. odontolytica* and *Actinomyces naeslundii* were detected in 10^9^ and 10^7^ colony-forming units per mL (CFU/mL), respectively, in addition to other anaerobic bacteria (Table 1). During the surgery and hospitalization, the patient received a four-week course of i.v. amoxicillin 3 × 1.2 mg. Before discharge, the patient received detailed practical instructions on how to maintain good oral hygiene. Since then, the patient appeared for control examinations monthly. At present – six months after the surgical intervention - the patient presents symptom-free, with no inflammation or denuded bone surface in the post-operative region.

**Table 1.** Summary of microbiological culture results from MRONJ necrotic bone samples for Cases 1–3

Case 1 MRONJ Stage 1–2		Case 2 MRONJ Stage 3		Case 3 MRONJ Stage 2	
Identified microorganisms	CFU/mL	Identified microorganisms	CFU/mL	Identified microorganisms	CFU/mL
***Schaalia odontolytoca***	**10** ^**9**^ ** CFU/mL**	***Actinomyces naeslundii***	**10** ^**9**^ ** CFU/mL**	***Actinomyces naeslundii***	**10** ^**9**^ ** CFU/mL**
***Actinomyces naeslundii***	**10** ^**7**^ ** CFU/mL**	***Schaalia odontolytoca***	**10** ^**9**^ ** CFU/mL**	***Schaalia odontolytoca***	**10** ^**9**^ ** CFU/mL**
*Veillonella parvula*	10^7^ CFU/mL	*Fusobacterium nucleatum*	10^7^ CFU/mL	*Parvimonas micra*	10^5^ CFU/mL
*Eikenella corrodens*	10^7^ CFU/mL	*Porphyromonas gingivalis*	10^7^ CFU/mL	*Prevotella buccae*	10^5^ CFU/mL
*Fusobacterium nucleatum*	10^7^ CFU/mL	*Filifactor alocis*	10^7^ CFU/mL	*Veillonella atypica*	10^5^ CFU/mL
*Fusobacterium mortiferum*	10^7^ CFU/mL	*Solobacterium moorei*	10^6^ CFU/mL	*Veillonella dispar*	10^5^ CFU/mL
*Clostridium beijerneckii*	10^6^ CFU/mL	*Eubacterium cellulosolvens*	10^5^ CFU/mL	*Gemella haemolysans*	10^4^ CFU/mL
*Solobacterium moorei*	10^6^ CFU/mL	*Peptostreptococcus stomatis*	10^5^ CFU/mL	*Enterobacter ludwigii*	< 10^3^ CFU/mL
*Camplyobacter recta*	10^6^ CFU/mL	*Veillonella rogosa*	10^5^ CFU/mL		
*Streptococcus anginosus*	< 10^3^ CFU/mL	*Streptococcus constellatus*	10^5^ CFU/mL		

*Actinomyces* spp. and related organisms are shown in **boldface**; MRONJ: medication-related osteonecrosis of the jaw; CFU/mL: colony-forming units per mililiters.

### Case 2

**Fig. 2.**
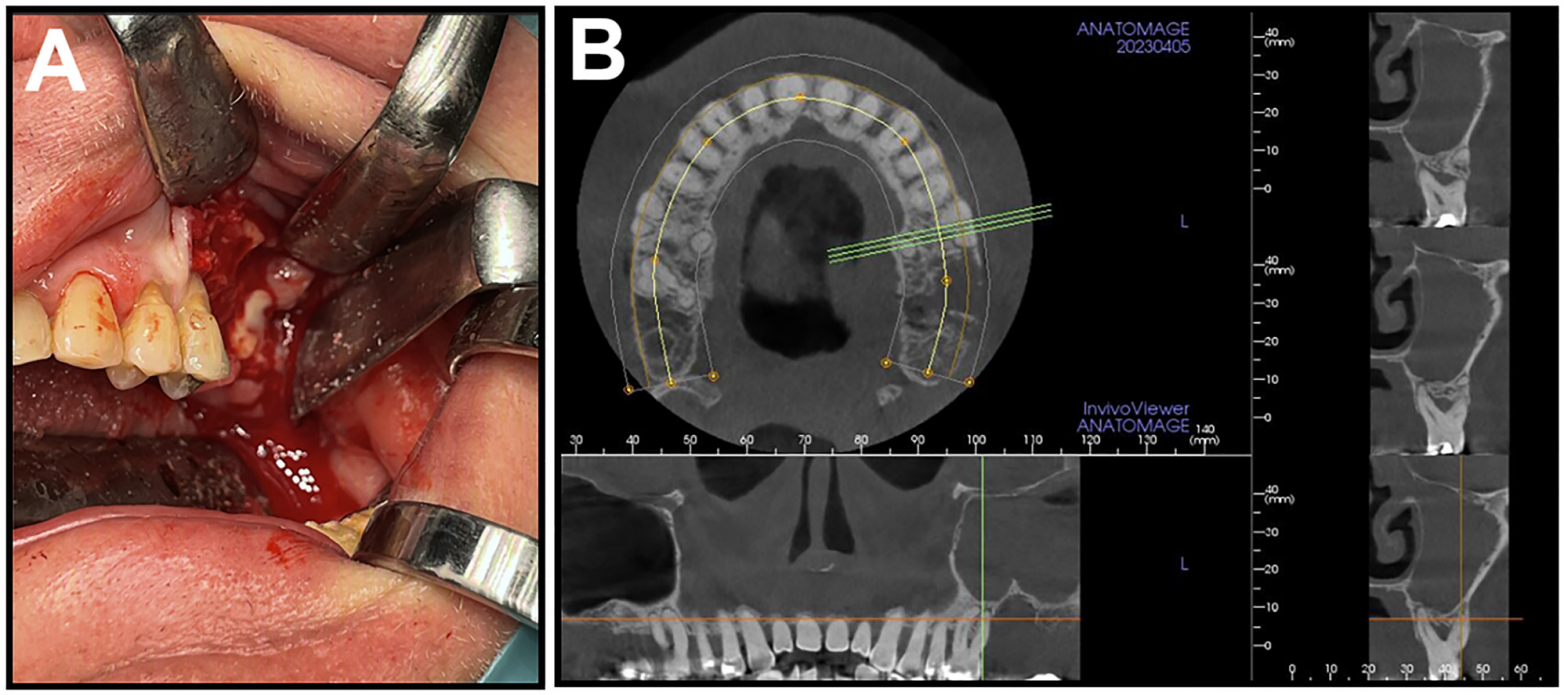
Clinical images corresponding to Case 2 **A:** Clinical presentation of the patient during surgery; **B:** Cone-beam CT (CBCT) image of patient before the surgical intervention

A 68-year-old male patient has undergone a prostate biopsy, due to elevated prostate-specific antigen (PSA) values during laboratory analyses; the biopsy confirmed a diagnosis of prostate adenocarcinoma (with a Gleason score 8 (5 + 3)). Computer tomography (CT) scans showed enlarged parailiac and paraaortic lymph nodes. Surgical treatment involved a radical prostate-vesiculectomy and extended pelvic lymphadenectomy. Following the surgery, radiation treatment (66/2 Gy) was administered. 5 months later, scintigraphy identified bone metastases in 4 ribs and in the left shoulder; subsequently, i.v. 4 mg zoledronic acid therapy was given every four weeks. One year later, scintigraphy confirmed the progression of bone metastases, which has led to a switch to 120 mg i.v. denosumab therapy monthly. Three years after the initiation of denosumab treatment, the 2.7. tooth fell out spontaneously, after which the patient presented at the Department of Dentistry, Oral and Maxillofacial Surgery with non-specific complaints in the left upper molar region. The clinical examination has shown that the surface of the post-extracted area was denuded, in addition, a fistula was also identified, with large amounts of pus being discharged from the fistula (Fig. 2). Cone-beam CT (CBCT) imaging showed a covered left sinus, an oroantral fistule and lytical lesions ranging from the molar till the premolar region (with a lenght of ∼2.5 cm), and tooth mobility of the 2.5 was also noted. The patient was diagnosed with Stage 3 MRONJ; the tissue destruction process was so advanced, that radical surgical intervention was deemed necessary by the clinicians. During the surgery, the necrotic bone lesion and the fistula were eliminated, during which, a huge amount of pus was detected in the sinus; subsequently, a Luc-Caldwell procedure was performed. Under sterile conditions, a part of the necrotized bone was sampled and placed in 1 mL sterile, pre-reduced BHI broth, and sent for processing at the Department of Medical Microbiology and Immunology, where culture was initiated immediately. Culture results showed – in addition to other anaerobic species – *A. naeslundii* and *S. odontolytica* as the most important isolates, with 10^9^ CFU/mL density for both species (Table 1). During hospitalization, the patient received i.v. amoxicillin 3 × 1.2 mg. Before discharging the patient, he was instructed on maintaining good oral hygiene and the advantages of smoking cessation. The antibiotic therapy of the patient was changed to *per os* amoxicillin 2 × 1 mg, for a duration of four weeks. Three months after the surgery, the patient had no symptoms at the control examination, the control CBCT showed a moderately covered sinus. Currently – nine months after the surgery – the patient is still without complaints, and no inflammation or denuded bone surface was noted in the post-operative region.

### Case 3

**Fig. 3.**
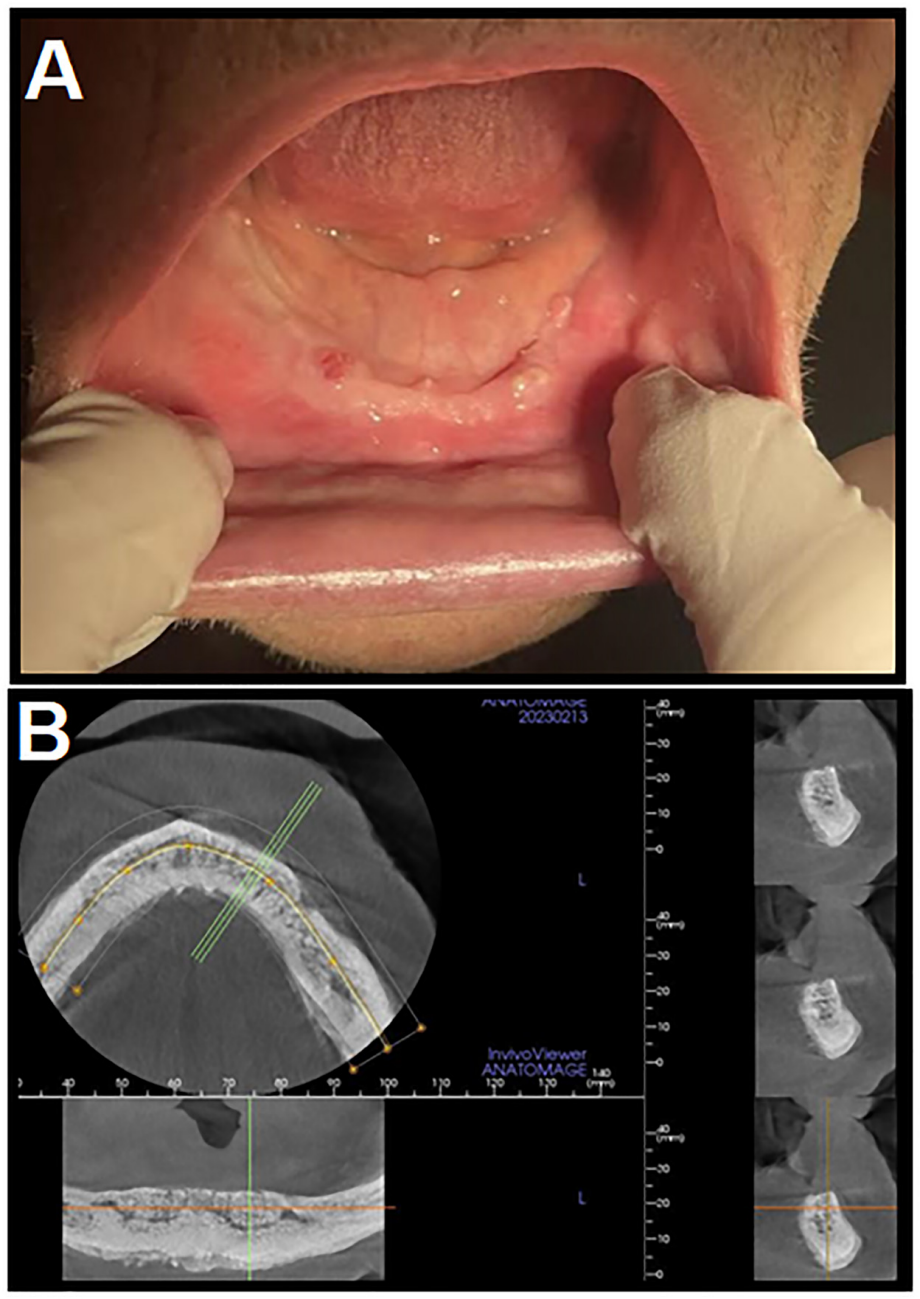
Clinical images corresponding to Case 3 **A:** Clinical presentation of the patient during surgery; **B:** Cone-beam CT (CBCT) image of patient before the surgical intervention

A 86-year old male patient was diagnosed with prostate cancer (with a Gleason score 7 (4 + 3)), for which, surgical therapy was chosen as primary treatment, involving a radical prostato-vesiculotomy with an extended pelvic lymphadenctomy. Following surgical treatment, endocrine deprivation therapy (ADT) and radiation treatment (66/2 Gy) were initiated. Control scintigraphies were negative up until the 50th month after the surgery, where bone metastasises were found in the lumbal and thoracic spinal region. Subsequently, i.v. 4 mg zoledronic acid therapy was given every four weeks, which was switched after three years to monthly 120 mg i.v. denosumab therapy. Two years after the initiation of denosumab treatment, the patient was hospitalized at the Department of Dentistry, Oral and Maxillofacial Surgery due to a submandibular abscess of the left side; at the time, the patient was edentulous, and was wearing a removable denture. A surgical incision was performed, and the patient received i.v. amoxicillin 3 × 1.2 mg for a week. There were no complications, and the patient's status was unremakrable at the following control visits, although the patient used his dentures only occasionally. However, 9 months after the surgery, denuded bone was observed around the two lower canines space, with pus discharge and severe inflammation. CT and single photon emission computed tomography (SPECT) CT imaging were performed to assess the intraforaminal necrotic bone lesion. The patient was diagnosed with Stage 2 MRONJ; during a follow-up surgery, the bone lesion was totally eliminated, and the surgical region was covered by platelet rich fibrin (PRF) and suture (Fig. 3). Under sterile conditions, a part of the necrotized bone was sampled and placed in 1 mL sterile, pre-reduced BHI broth, and sent for processing at the Department of Medical Microbiology and Immunology, where culture was initiated immediately. Culture results showed a mixed anaerobic culture, with *A. naeslundii* and *S. odontolytica* being the dominant isolates, with 10^9^ CFU/mL density for both species (Table 1). The patient received i.v. amoxicillin 3 × 1.2 mg for a week, which was then switched to *per os* amoxicillin 2 g/die for five weeks. Six months after the surgery, the patient is asymptomatic, and no denuded bone surface could be observed.

## Methods

### Sample processing and bacterial identification

Samples obtained from respective patients were processed at Department of Medical Microbiology and Immunology, in accordance with International guidelines in routine microbiology. Samples taken were sent to the laboratory immediately after collection, and were processed within 1 h of receipt. The samples were suspended in 1 mL BHI broth (Oxoid, Basingstoke, United Kingdom), gently dispersed, and diluted (10^−1^ – 10^−9^) in pre-reduced BHI broth [39]. Then, 100 µL of each dilution were plated on selective and non-selective media. For anaerobic culture, Schaedler agar (containing horse blood 5% V/V, haemin and Vitamin K_1_; bioMérieux, Marcy l'Etoile, France) plates were used; these cultures were incubated in in an atmosphere of 90% N_2_, 5% H_2_ and 5% CO_2_ in an anaerobic environment (Concept 400 anaerobic incubator, Biotrace International Plc., UK) for 5–14 days at 37 °C [40].

Identification of the isolated was carried out using matrix-assisted laser desorption/ionization time-of-flight mass spectrometry (MALDI-TOF MS); the microFlex LT Biotyper instrument (Bruker Daltonics Gmbh., Bremen, Germany), the MALDI Biotyper RTC 3.1 software (Bruker Daltonics, Germany) and the MALDI Biotyper Library 3.1 were used for spectra analysis. Sample preparation procedures and the technical details of the MALDI measurements were described in detail elsewhere [41]. Based on concesus criteria, genus-level identification of bacteria isolates was deemed reliable if the log(score)≥1.7, while for reliable species-level identification, a log(score)≥2.0 was needed [42].

### Ethical considerations

The study was conducted in accordance with the Declaration of Helsinki and national and institutional ethical standards. Ethical approval for the study protocol was obtained from the Human Institutional and Regional Biomedical Research Ethics Committee, Medical School, University of Pécs (registration number: 9593-PTE 2023). Written informed consent was obtained from the patients involved in this study.

## Discussion

*Actinomyces* species and ALOs are important members of the commensal oral flora, however, they may act as facultative pathogens causing cervicofacial actinomycosis, especially when protective anatomical barriers break down (e.g., due to trauma, injuries), allowing for the entry of these bacteria into normally sterile anatomical regions [26, 43]. These infections are often polymicrobial in nature, involving other members of the oral microbiota. In the present paper, we aimed to provide additional, microbiological culture-supported evidence, supporting the hypothesis that *Actinomyces* spp. may play an important pathogenic role in the development of MRONJ and the resulting bone necrosis. In our case series, all three patients presented with similar underlying conditions and anamnestic data, and have received antiresorptive medications (either bisphosphonates or a RANK-L inhibitor, or both) to prevent the occurrence or progression of bone metastases, secondary to prostate cancer. Nevertheless, a few years into antiresorptive drug therapy, varying stages of MRONJ was identified in the mentioned patients; in all three cases, quantitative culture of the necrotic bone samples yielded a complex microbiota, dominated by *Actinomyces* and *Schaalia* spp. with high colony counts. Subsequently, all patients were treated with a combination of surgical intervention and antibiotic therapy (still the mainstay of anti-*Actinomyces* treatment, which is often characterized by long term – 1–6 months – drug therapy, to avoid relapse [26, 44]), where favourable clinical responses were seen is all cases.

With the ageing of the population, and a higher number of individuals affected by underyling diseases and multimorbidity, the prevalence of patients receiving antiresorptive drugs is expected to increase, which will undoubtedly lead to an increased incidence of MRONJ cases as well. Therefore, dentists must have awareness related to the adverse events and safety concerns associated with long-term use of antiresorptive medicines [45]. Based on recent studies, 0–27.5% of individuals receiving antiresorptive medications (especially ones affected by cancer) develop MRONJ [46]. According to a retrospective database analysis by Kotán and colleagues, 0.1% of patients developed BRONJ as a result of receiving bisphosphonates (with differences identified also among the drugs administered); the incidence of BRONJ when the drugs were given for malignant indications was 0.9%, while 0.1% in case on a non-cancer diagnosis [47]. Nevertheless, the MRONJ-risk associated with denosumab should not go underestimated; according to the study of Nashi and colleagues, denosumab-therapy accounted for 30% of overall MRONJ, while this ratio was 58% in individuals receiving high-dose antiresorptive treatment [48].

*Actinomyces* species and ALOs have limited number of “classical” virulence determinants (e.g., tissue-dissolving enzymes, exotoxins), rather, they possess factors relevant in the protection against and subversion of the host's immune response [25, 49, 50]. These factors include the formation of extracellular and cell-associated polysaccharides (which are relevant for biofilm-formation and the occurrence of the branching, dense, bacterial aggregates often found in clinical samples) that provide protection against phagocytes, antibodies and toxicity of metal ions, cell wall peptidoglycan (which will induce inflammatory cytokines, such as IL-1, IL-6 and TNF-α, and affect osteoclastogenesis), type I (relevant in binding to proline-rich proteins in the saliva, and to collagen in bone tissue) and type II (relevant in co-aggregation) fimbriae, and urease-enzyme production, among others [49–52]. These determinants may enhance antagonistic processes to tissue healing and recovery, and facilitate the binding of bacterial cells to exposed bone in the oral cavity. Increasing number of case reports and clinical studies suggest that *Actinomyces* spp. are not passive colonizers found in necrotic tissue, instead, they have a direct etiological role in causing bone necrosis. If the “infection hypothesis” is valid, it may have significant consequences in the preventative and therapeutic strategies associated with this disease [53]. The accumulation of recent studies all point to the direction that *Actinomyces* contributes to the inflammatory processes causing bone necrosis, or at the very least, use of antiresorptive or anti-antiangiogenesis drugs facilitate their invasion into deeper layers of bone tissue [54].

Unfortunately, at present, the evidence available is inconclusive, therefore, the conundrum of “chicken or the egg” remains to be solved in the context of MRONJ. Heterogeneity in the methods used to detect the presence of *Actinomyces* spp. (i.e. classical qualitative or quantitative culture, histology, polymerase-chain reaction [PCR], sequencing technologies) has brought on markedly different results, further complicating the matter [26]. Additionally, *Actinomyces* spp. and ALOs have long generation times, even among strict anaerobes, thus, if adequate time (5–14 days) is not alloted for culturing these microorganisms, traditional culture may easily miss them [25, 55]. Panya and colleagues highlighted the relevance and much-needed sensitivity of molecular biological methods in the detection of *Actinomyces* spp. from MRONJ-associated samples [56]: in a cohort of ninety-five patients with varying stages of MRONJ, they have utilized classical anaerobic culture methods with MALDI-TOF MS, PCR, and sequencing technologies to detect the microrganisms of interest. While 53/95 cases were positive for *Actinomyces* spp. by both culture and PCR, and additional 35/95 cases were detected only by PCR alone. Out of 101 oncological patients with histologically-confirmed MRONJ at Padova University Hospital, Cerrato and colleagues reported the presence of *Actinomyces* spp. infection in necrotic bone in 83 (82.2%) cases [57]. In the decade-long (2005–2014) of Russmuller and colleages, *Actinomyces* spp. was identified (either with culture or histology) in 94.6% of elderly patients (*n* = 111) with histologically-confirmed MRONJ in Vienna, Austria [58]. The association of cervicofacial actinomycosis and oral-health related underlying conditions was highlighted by Ibrahim and colleagues, where 93.5% of samples PCR-positive for *Actinomyces* spp. originated from either MRONJ or IORN patients, further establishing the association between these microorganisms and impacted bone in disease progression [59]. Similarly, Hansen and colleagues detected *Actinomyces* spp. in the samples of 20 out of 31 IORN patients, using histology and a semi-nested PCR [60]. Furthermore, the relevance of adequate histological workup, and clinician-pathologist consultation in the detection of *Actinomyces* spp. from MRONJ patients was shown by Brody and colleagues: using a validated, triplicate stain methodology, *n* = 112 previously hematoxylin-eosin (HE)-stained specimens a clinical diagnosis of MRONJ were re-assessed. While the initial histological evaluation (when the aim was not specifically to look for *Actinomyces* spp.) yielded only a ∼9% positivity, reassessment with triple staining (validated for the histological identification of *Actinomyces* infection) resulted in a >93% positivity rate [61].

In clinical practice, the principal aim is the prevention of MRONJ, i.e. to minimize the risk factors for the development of the condition, as the disease course and therapeutic success is often unpredictable [15, 62]. In addition, the advanced stages of the disease considerably affect the quality of life of patients. Nevertheless, many cases of MRONJ are only being identified in later stages of the illness, especially in older, male patients, whose attendance at dental checkups is notoriously low. Preventive strategies include extensive patient education and instruction, focused dental screening of individual receiving bisphosphonates or denosumab, elimination of existing oral diseases and the maintenance of good oral hygiene prior to initiating pharmacological treatment with medicines associated with MRONJ, and continuous check-ups by a dentist [15, 26, 62, 63]. For high-risk individuals (e.g., patients receiving antiresorptive drugs or radiotherapy due to a malignancy) cessation of antiresorptive treatment should be consulted after extensive oral surgery, and should only be re-initiated if the proper mucosal coverage and healing over the surgical site is observed. Avoidance of surgical trauma and infection to the jawbones considerably lowers the risk of MRONJ. On the other hand, once a MRONJ-related lesion develops, its management depends on the stage of the disease, the size and localization of the lesion, in addition to the patient's underlying characteristics (pharmacological treatment, comorbidities) [15, 26, 62, 63]. If there is clinical suspicion of *Actinomyces* spp. involvement, co-operation of the clinician and microbiologist is crucial – as demonstrated by the outcomes of our cases – as it considerably improves the chance of correct diagnosis, timely, actinomycosis-specific treatment, and overall, it may lead to improved outcomes. Consultation with the microbiology laboratory ensures clear instructions on sample procurement, the use of valid culture methods, longer culture time and and/or molecular biological techniques [25, 26, 64]. Finally, therapeutic success of MRONJ (depending on the stage of the disease) largely depends on the use of appropriate, long-term antibiotic therapy (which most commonly entails the administration of penicillin-derivatives, like amoxicillin and ampicillin; alternatively, macrolides or clindamycin may also be relevant in case of pencillin-allergy) to eradicate the *Actinomyces* infection and to prevent relapse [65].

## Funding sources

No financial support was received for this study.

## Authors' contributions

Z.K., Á.B. and E.U. conceived and designed the study; Z.K., Á.B. were involved in the treatment of patients and clinical data collection; B.P. and E.U. performed microbiological analyses; Z.K. wrote the initial version of the manuscript; M.G. and E.U. wrote and revised the full paper. All authors have read and agreed to the published version of the manuscript.

## Conflict of interest

The authors declare no conflict of interest, monetary or otherwise. The authors alone are responsible for the content and writing of this article.

## Supporting information

None. All data generated during the study are presented in this paper.
